# Assessment of Macular Thickness in Healthy Eyes Using Cirrus HD-OCT: A Cross-Sectional Study

**Published:** 2016

**Authors:** Mohammad Rasoul SABOURI, Ehsan KAZEMNEZHAD, Vahideh HAFEZI

**Affiliations:** 1 Guilan University of Medical Sciences, Rasht, Iran

**Keywords:** Macular thickness, Healthy subject, Cirrus HD-OCT, Rasht

## Abstract

We aimed to determine normal macular thickness using Cirrus high definition optical coherence tomography. In this cross-sectional survey, 112 subjects were selected using random sampling from the Rasht telephone directory. All subjects underwent complete eye examinations. Both eyes of each patient were evaluated. The creation of a macular thickness map using a macular cube 512 × 128 combo was optional. The average thickness of the retina was determined in 9 Early Treatment Diabetic Retinopathy Study (ETDRS) regions. To assess reproducibility and system reliability, the thickness of the retina was measured up to 5 times in 10 healthy subjects. The coefficient of variation was then calculated for each individual. The coefficient of variation of macular thickness within 1 mm of the center was 0.15 - 1.33%. The means and standard deviations of central subfield thickness (CST), macular thickness (MT), and macular volume (MV) were 245.44 ± 20.39 µm, 277.9 ± 12.0 µm, and 9.98 ± 0.43 mm^3^, respectively. The mean CST (P < 0.0001), MT (P = 0.038), and MV (P = 0.030) were significantly higher in men than in women. In addition, regardless of age or sex, macular thickness increased when moving from within 1 mm of the center to 3 mm and 6 mm away from the center, so that the upper 3 mm (S3) was the thickest region, and the temporal 6 mm (T6) was the thinnest region in the ETDRS regions. The mean MT of healthy subjects was 280.67 ± 12.79 µm in men and 276.63 ± 11.61 µm in women. Therefore, the macula is significantly thicker in men than in women (P = 0.038).

## Introduction

Macular edema is a common cause of visual impairment, and macular thickness is significantly associated with visual acuity (1). Increased retinal thickness due to fluid accumulation is common in ophthalmological diseases, such as diabetic retinopathy, age-related maculopathy, central serous chorioretinopathy, and venous occlusions. Therefore, knowledge of macular thickness is important when assessing pathological cases. Conventional assessments of macular edema or thickness using techniques such as fundus photography and fluorescein angiography are qualitative and have no sensitivity to slight changes in thickness. Optical coherence tomography (OCT) devices are used for quantitative assessment of retinal thickness. These devices provide a non-invasive and non-contact real optical biopsy of the posterior segment (1). Since its introduction in the last decade, OCT technology has undergone great advances in the number of A-scans per second (acquisition speed) and axial resolution. For example, Stratus devices (ST-OCTs) are capable of performing 400 A-scans per second with axial resolutions of 8-10 µm (2), and the numbers of A-scans per second are much higher (more than 20,000 per second) when lower axial resolutions are used (5-7 µm). This leads to more accurate imaging of the retina (3). As a result, the use of different OCT devices may lead to different data regarding retinal thickness in normal individuals. Studies have shown that central retinal thicknesses measured in normal individuals using Spectralis devices are significantly higher than those measured using Stratus devices (4, 5). Some reports indicate differences as large as 50-70 µm (6, 7).

One reason for these differences is that the anterior-posterior boundaries of the retina are different in these devices. For example, in ST-OCT and spectral domain OCT (SD-OCT) devices, the boundaries are determined from the inner membrane layer to the inner/outer segment and from the internal limiting membrane to the pigmented layer, respectively (1, 5, 6). It should be noted that the segmentation algorithm used in SD-OCT devices is also different from that used in other devices. For instance, the retinal outer boundary in Cirrus HD-OCT and Topcan-SD devices is the inner boundary of the pigmented layer (1, 7). This boundary is the outer boundary of the pigmented layer in HRL +OCT devices. In fact, even normal data obtained using Cirrus HD-OCT devices may differ between studies. For example, in 5 separate studies, the mean central subfield thickness of the macula was reported as: 262 ± 23 µm in 192 eyes (8), 276 ± 17 µm in 40 eyes (5), 257.6 ± 19.6 µm in 50 eyes (9), 300 µm in 28 eyes (4), and 266.2 ± 22.7 µm in 50 eyes (7). The different values suggest that the range of normal macular thickness is imprecisely measured. Other factors leading to significant differences in macular thickness include race, age, and gender. In addition, normal maculae may have different thicknesses than maculae affected by disease. We therefore conducted this study to evaluate normal macular thickness and its variations according to age and sex using Cirrus HD-OCT (Carl Zeiss Meditec, Inc., Dublin) in Rasht.

## MATERIALS AND METHODS

In this study, 132 random individuals were selected from the Rasht telephone directory using systematic random selection. The selected individuals were then contacted by telephone, and those in the 20–40-year, 41–60-year, and over 60-year age groups were invited to participate in the study. The subjects were then referred to the clinic. At that point, they underwent the macular thickness measurements if they met the inclusion criteria. All study subjects signed informed consent forms before the ocular examinations. This study was approved by the ethics committee of the Guilan University of Medical Sciences (GUMS) under ethics approval number 1920251608. Of the 132 subjects, 112 were eligible to participate in the study. The subjects participating in the study were classified into age groups of 20-40, 41-60, and over 60 years. All participants underwent complete ophthalmological examinations to assess retinal diseases and glaucoma using + 20 and + 90 lenses and applanation tonometry by a surgeon. All OCT scans were performed by a single operator and both of each subject's eyes were tested following pupil dilation using 1% Mydriacyl, Sina Darou Lab. Co., Iran.

We used a macular thickness map protocol with a macular cube 512 × 128 combo. We obtained mean retinal thickness measurements in 9 Early Treatment Diabetic Retinopathy Study (ETDRS) regions. Only images whose signal strengths were in the green range (more than 50%) and had good quality (no artifact) were selected. Individuals with a retinal disease or glaucoma, those with intraocular pressures (IOPs) > 21 mmHg, a history of surgery or laser surgery, VA (Visual Aquity) < 20/20, or refractive errors greater than ± 5 were excluded from the study. The data entered into the SPSS version 16. We used the Cirrus device (Cirrus HD-OCT (Carl Zeiss Meditec, Inc., Dublin) to determine normal macular thickness in individuals separated by age and sex with a confidence interval of 95%. We measured mean macular thickness (MT), macular volume (MV), and central subfield thickness (CST). Independent T-tests and analyses of variance (ANOVAs) were used to compare the mean CST, MT, and MV in individuals grouped by sex and age. *P*-values less than 0.05 were considered significant in the two-sided tests.

Intra-class correlation coefficients (ICCs) were used to evaluate the intra-observer reliability of macular thickness measurements by Cirrus-HD OCT. The ten subjects used in this experiment had a mean of age of 53.4 ± 15.0 years. Each subject was evaluated five times. When both eyes from each subject were measured, we obtained a mean ICC of 0.95 and a 95% confidence interval of 0.888-0.986. These results indicate that our observations had high reliability.

Scientific Definitions

**Nine ETDRS Regions**

**Figure 1:**
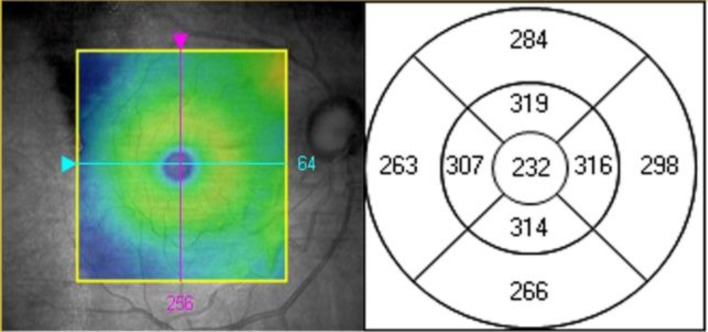
Nine Early Treatment Diabetic Retinopathy Study (ETDRS) regions.

The ETDRS study group defined 9 regions used to evaluate changes in macular thickness during the course of diabetic retinopathy. The regions are located in three rings with diameters of 1, 3, and 6 mm. The 1 mm ring contains a 1 mm perifovea ring called the Central Retinal Subfield. The 3 and 6 mm rings are located 3 and 6 mm from the first ring, respectively. Each ring is divided into 4 quadrants: superior, inferior, temporal, and nasal. We have designated these regions S, I, T, and N for simplicity. For example, S_3_ and T_6_ represent the upper region in the 3 mm ring and the temporal region in the 6 mm ring, respectively (Figure 1).

## RESULTS

The coefficient of variation of mean macular thickness in the 10 subjects, who underwent five measurements each in both eyes, was 0.15-1.37%. This indicates that the reproducibility of the device is adequate. One-hundred and twelve healthy individuals (21.4% men, n = 24; 78.6% women, n = 88) participated in this study. The participants had a mean age (± standard deviation) of 49.7 ± 12.1 years. The youngest and oldest individuals were 20 and 73 years old, respectively. The age distribution of the subjects was as follows: 16.1%, 63.4%, and 20.5% in the 20–40-year, 41-60-year, and over 60-year age groups, respectively.

Table 1 shows CST, MT, and MV thickness in 112 subjects, as well as mean thickness in the 9 ETDRS regions in the 224 eyes. Mean CST, MT, and MV in the total population were 245.44 ± 20.39 µm, 277.4 ± 11.95 µm, and 9.98 ± 0.43 mm^3^ (confidence intervals = 95%), respectively. It should be noted that MT increases when moving from the 1 mm center region of the macular toward the 3 and 6 mm regions, so that the superior zone of the 3 mm region and the temporal zone of the 6 mm region are the thickest (320.26 ± 14.43 µm) and thinnest (260.18 ± 14.23 µm) parts of the macula, respectively. 

As shown in Table 2, comparing MT, CST, MV, and other thicknesses in men and women show that CST (P < 0.0001), MV (P = 0.03), and MT (P = 0.038) are higher in men than in women.

**Figure 2:**
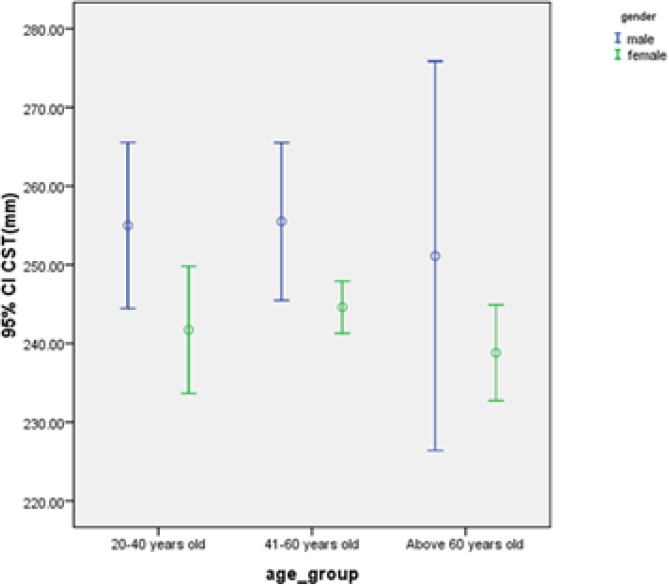
Comparison of 95% confidence intervals of mean CST according to age group

We calculated Pearson correlation coefficients to determine whether aging is associated with changes in macular thickness. Our results indicate that there is a minor decrease in thickness in most regions, although this difference is not significant. In addition, MT is decreased by 0.23 µm with every 10-year increase in age, although this reduction was also non-significant (P = 0.728). Table 3 shows CST, MT, and MV thickness in different age groups. We assessed differences in the thicknesses of the 9 macular regions using ANOVA in the 3 age groups (20-40, 41-60, and over 60 years). There were no statistically significant differences between the 3 age groups. The central macular thickness values are shown in Figure 2.

**Table 1:** Average and the sectorial retinal nerve fiber layer thickness (in µm) in healthy eyes in Rasht city

	**N**	**Mean**	**Std. deviation**	**Median**	**Minimum**	**Maximum**
**CST (** **µ** **m)**	224	245.45	20.39	246.00	193.00	311.00
**MV (mm** ^3^ **)**	224	9.99	.43	10.00	9.00	11.20
**MT (** **µ** **m)**	224	277.50	11.96	278.00	250.00	312.00
**C**	224	245.42	20.42	246.00	193.00	311.00
**N3**	224	319.70	15.04	320.00	277.00	360.00
**N6**	224	293.99	15.16	296.00	231.00	333.00
**T3**	224	305.99	15.61	305.50	223.00	355.00
**T6**	224	260.18	14.24	261.00	229.00	311.00
**S3**	224	320.27	14.44	321.00	288.00	367.00
**S6**	224	276.77	12.76	277.00	246.00	318.00
**I3**	224	316.87	15.93	317.00	273.00	383.00
**I6**	224	267.44	13.54	267.50	235.00	306.00

Abbreviations: CST = central subfield thickness, MV = macular volume, MT = macular thickness, C = central, N3 = nasal inner macula, N6 = nasal outer macula, T3 = temporal inner macula, T6= temporalouter macula, S3 = superior inner macula, S6 = superior outer macula, I3 = inferior inner macula, I6 = inferior outer macula.

We also compared macular thicknesses of the 9 regions between the right and left eyes. There were no statistically significant differences between the sizes of the right and left eyes, as determined using paired T-tests (Table 4).

## DISCUSSION

Reproducibility in tissue measurements is a key advantage of using OCT devices. We therefore first assessed the reproducibility of the measurements obtained using the Cirrus device in this study. In this cross-sectional survey, the coefficient of variation for the device was 0.15 - 1.37%, which suggests good reproducibility. We found that men had significantly higher CST (P < 0.0001), MV (P = 0.03), and MT (P = 0.038) values than women. Kakinoki et al. initially determined devices reproducibility when measuring normal macular thickness using Cirrus HD and SD-OCT, and compared these values to those obtained using time domain OCT devices. The reproducibility coefficient of Cirrus HD was reported to be 0.2-1.3% (mean of 0.66%), which indicates high power (9). In this study, the mean thicknesses of the CST, MT, and MV were found to be 245.44 ± 20.39 µm, 277.4 ± 12.0 µm, and 9.98 ± 0.43 mm^3^, respectively. These values were found to be higher in men than in women, although we did not observe any significant differences in these values in the different age groups. In addition, regardless of age or sex, macular thickness increased when moving from within 1 mm of the center to the 3 and 6 mm regions, so that the central region and the superior zone of the 3 mm ring were the thinnest and the thickest regions, respectively. The temporal zone of the 6 mm region was the thinnest peripheral section. We also found that mean MT was not significantly different between the right and left eyes.

In a study on 192 eyes from 192 healthy individuals aged between 20 and 90 years using the Cirrus HD device. They reported that the CST, MT, and MV were 262.4 ± 22.8 µm, 281 ± 14.5 µm, and 10.1 ± 0.6 mm^3^, respectively. In Liu et al.’s study, the mean CST did not change with increasing age, although the mean MT and MV decreased significantly with age (P < 0.0001). In addition, the mean MT was lower in women than in men. The authors of that study suggested that the 1 mm region and the superior zone of the 3 mm region are the thinnest and thickest areas of the macula, respectively, and that the thinnest peripheral section of the macula is the inferior portion of the 6 mm region. Although we found that quantitative changes in macular thickness occur with increasing age (decrease of 0.23 µm with each 10 years of increasing age), these changes have no clinical importance. The Liu et al. study indicates that CST thickness is the only measure that does not undergo changes with age, as MT and MV thickness decreased significantly with increasing age. These results are inconsistent with our findings. In a study conducted by Kakinoki et al., normal macular thickness in 50 eyes from 50 healthy subjects with a mean age of 49.9 ± 18.0 years and equal sex distribution was assessed using the Cirrus HD device. The authors found a mean macular thickness of 257 ± 19.6 µm. There was no correlation between age and changes in macular thickness (9), which is consistent with our results.

**Table 2:** Average and the sectorial retina nerve fiber layer thickness (in µm) distribution by sex

**Parameters**	**N**	**Mean**	**Std. Deviation**	**P value**
**CST (µm)**				0.000
male	48.00	254.67	24.90	
female	176.00	242.93	18.27	
**MV (µm)**				0.030
male	48.00	10.10	0.45	
female	176.00	9.95	0.42	
**MT (µm)**				0.038
male	48.00	280.67	12.79	
female	176.00	276.63	11.61	
**C**				0.000
male	48.00	254.67	24.90	
female	176.00	242.89	18.30	
**N3**				0.000
male	48.00	327.67	14.65	
female	176.00	317.52	14.44	
**N6**				0.200
male	48.00	296.48	14.89	
female	176.00	293.31	15.21	
**T3**				0.000
male	48.00	315.69	15.31	
female	176.00	303.34	14.66	
**T6**				0.004
male	48.00	265.40	15.59	
female	176.00	258.76	13.55	
**S3**				0.000
male	48.00	327.25	14.63	
female	176.00	318.36	13.83	
**S6**				0.477
male	48.00	277.94	14.63	
female	176.00	276.45	12.23	
**I3**				0.000
male	48.00	324.33	17.79	
female	176.00	314.83	14.80	
**I6**				0.297
male	48.00	269.25	14.63	
female	176.00	266.94	13.23	

Abbreviations: CST = central subfield thickness, MV = macular volume, MT = macular thickness, C = central, N3 = nasal inner macula, N6 = nasal outer macula, T3 = temporal inner macula, T6 = temporal outer macula, S3 = superior inner macula, S6 = superior outer macula, I3 = inferior inner macula, I6 = inferior outer macula

**Table 3:** Average and sectorial retina nerve fiber layer thickness (in µm) in different age groups

	**N**	**Mean**	**Std. Deviation**	**P**
**CST (µm)**				0.229
20-40 years old	36	245.42	19.44	
41-60 years old	142	246.91	20.36	
Above 60 years old	46	240.96	21.01	
Total	224	245.45	20.39	
**MV (µm)**				0.427
20-40 years old	36	9.96	0.33	
41-60 years old	142	10.01	0.44	
Above 60 years old	46	9.92	0.45	
Total	224	9.99	0.43	
**MT (µm)**				0.336
20-40 years old	36	276.92	9.35	
41-60 years old	142	278.32	12.38	
Above 60 years old	46	275.39	12.38	
Total	224	277.50	11.96	
**C**				0.219
20-40 years old	36	245.42	19.44	
41-60 years old	142	246.89	20.36	
Above 60 years old	46	240.85	21.10	
Total	224	245.42	20.42	
**N3**				0.611
20-40 years old	36	320.81	11.83	
41-60 years old	142	320.03	15.55	
Above 60 years old	46	317.80	15.80	
Total	224	319.70	15.04	
**N6**				0.571
20-40 years old	36	295.25	9.58	
41-60 years old	142	294.32	15.56	
Above 60 years old	46	291.98	17.40	
Total	224	293.99	15.16	
**T3**				0.536
20-40 years old	36	304.89	10.96	
41-60 years old	142	306.86	16.90	
Above 60 years old	46	304.15	14.60	
Total	224	305.99	15.61	
**T6**				0.213
20-40 years old	36	256.58	11.85	
41-60 years old	142	261.23	14.64	
Above 60 years old	46	259.78	14.49	
Total	224	260.18	14.24	

Abbreviations: CST = central subfield thickness, MV = macular volume, MT = macular thickness, C = central, N3 = nasal inner macula, N6 = nasal outer macula, T3 = temporal inner macula, T6 = temporal outer macula, S3 = superior inner macula, S6 = superior outer macula, I3 = inferior inner macula, I6 = inferior outer macula

**Table 4:** Correlations between the age and retina nerve fiber layer thickness (in μm) in all participants

**Correlations**	**Age**	
**CST (** **µ** **m)**		
Pearson Correlation	-0.019
P	0.779
**MV (mm** ^3^ **)**	
Pearson Correlation	-0.019
P	0.779
**MT (** **µ** **m)**	
Pearson Correlation	-0.023
P	0.728
**C**	
Pearson Correlation	-0.021
P	0.756
**N3**	
Pearson Correlation	-0.025
P	0.711
**N6**	
Pearson Correlation	-0.028
P	0.674
**T3**	
Pearson Correlation	0.002
P	0.975
**T6**	
Pearson Correlation	0.088
P	0.191
**S3**	
Pearson Correlation	0.046
P	0.496
**S6**	
Pearson Correlation	0.004
P	0.952
**I3**	
Pearson Correlation	-0.014
P	0.830
**I6**	
Pearson Correlation	-0.028
P	0.675

Abbreviations: CST = central subfield thickness, MV = macular volume, MT = macular thickness, C = central, N3 = nasal inner macula, N6 = nasal outer macula, T3 = temporal inner macula, T6 = temporal outer macula, S3 = superior inner macula, S6 = superior outer macula, I3 = inferior inner macula, I6 = inferior outer macula

Wolf-Schnurrbusch et al. conducted another study to determine normal macular thickness in healthy subjects using six different OCT devices. In that study, the mean CST of macula in 40 eyes from 20 healthy subjects over 18 years of age was found to be 276 ± 17 µm in the right eye and 277 ± 21 µm in the left eye when using the Cirrus HD. There was no significant difference in the thickness of the central region between the right and left eyes (5). These results are also consistent with our study. Legarreta et al. measured normal macular thickness using the Cirrus HD in 50 eyes from 50 healthy subjects of both sexes with almost equal numbers ranging in age from 20 to 68 years. The mean central macular thickness was found to be 266.2 ± 22.7 µm. The authors also found that the area of the macula within 3 mm of the center is the thickest, and that macular thickness decreases when moving from the 3 mm region to the 6 mm region. The results of this study were inconsistent with our study. In a cross-sectional study, Choovuthayakorn et al. measured macular thickness in 368 healthy Thai subjects using a Spectralis SD-OCT device. Even though the authors did not use a Cirrus SD-OCT in their study, the measured mean CST was 265.05 ± 18.62 and 252.84 ± 17.55 µm in men and women, respectively. As in our study, these values were significantly different. The authors found no significant differences in macular thickness between individuals with different ages. These findings are consistent with the current study.

In conclusion, macular thickness has a wide range. This is why measured thicknesses of normal maculae using a single device in different studies vary according to sex and age. In this study, the mean values of MT obtained using the Cirrus HD in men in Rasht was found to be 277 - 285 µm (95% CI). We found a statistically significant difference in macular thickness between men and women. Liu et al. from California found a mean MT of 281.3 ± 14.5 µm, and also observed significant differences between the two sexes. Kakinoki et al. from Japan found a mean MT of 257 ± 19.6 µm, while Wolf-Schnurrbusch et al. reported a mean MT of 277 ± 21 µm. Considering the 95% confidence intervals, the observed average macular thickness in our study (277 - 285 µm) is similar to that observed in the Liu study (267 - 295 µm). However, there is a statistically significant difference in macular thickness between our study and Kakinoki’s study (250 - 264 µm) and Wolf’s study (272 - 277 µm) (P < 0.05). This indicates that normal macular thickness varies in individuals from different communities. The results of this study can thus only be used to evaluate individuals from the north of Iran, but not those from other provinces.
